# Analysis of electric cigarette liquid effect on mouse brain tumor growth through EGFR and ERK activation

**DOI:** 10.1371/journal.pone.0256730

**Published:** 2021-09-08

**Authors:** Hyung Joon Kwon, Young Taek Oh, Saewhan Park, Sung Soo Kim, Jinju Park, Jinlong Yin, Jun Hee Hong, Chan Il Kim, Haseo Ryu, Jong Bae Park, Min Kyung Lim

**Affiliations:** 1 Department of Cancer Control and Population Health, National Cancer Center Graduate School of Cancer Science and Policy, Goyang, South Korea; 2 Division of Clinical Research Institute and Hospital, National Cancer Center, Goyang, Republic of Korea; 3 Department of Cancer Biomedical Science, National Cancer Center Graduate School of Cancer Science and Policy, Goyang, South Korea; 4 Department of Social and Preventive Medicine, Inha University College of Medicine, Incheon, South Korea; University of Messina, ITALY

## Abstract

**Introduction:**

Recently, electric cigarettes with liquid (e-liquid) were introduced as an alternative to tobacco smoking. They were promoted as possible cessation aids and were considered to be potentially less harmful than traditional tobacco-based cigarettes. However, there is little information on the toxicants present in e-liquids and their possible carcinogenic effects.

**Methods:**

Western blot analysis was performed to identify the protein levels of cancer progression related signal transducers. Patient-derived brain tumor cells (CSC2) were injected into mouse brains and tumor growth was then observed by performing magnetic resonance imaging (MRI) and hematoxylin and eosin (H&E) staining of the whole brain. Immunohistochemistry (IHC) staining and Immunofluorescence staining were performed to study the expression of pEGFR and pERK.

**Results:**

Western blotting revealed that e-liquids increased pEGFR and pERK expression in a dose dependent manner. Animal experiments revealed that the e-liquid treated group had accelerated tumor growth and poor prognosis compared to the vehicle group. Histological staining showed activation of pEGFR and pERK in the e-liquid treated group.

**Conclusion:**

Our study revealed that e-liquid activates pEGFR and pERK, leading to accelerated brain tumor growth and poor prognosis.

## Introduction

Tobacco use is a major threat to health worldwide and several epidemiological studies attribute tumorigenesis and tumor progression to the use of tobacco [1–3]. Different types of nicotine delivery systems have been developed by tobacco companies as a measure to avoid tobacco use [4,5]. Recently, electric cigarettes (e-cigarettes) were introduced as a less harmful alternative compared to traditional cigarette [6,7]. Moreover, earlier studies have shown lower amounts of toxicants and carcinogens, such as nicotine and TSNAs (Tobacco-specific nitrosamines; group 1 carcinogen), in e-cigarette liquid (e-liquid) and emission compared to the toxicants and carcinogens in the emission from conventional cigarettes [8–11]. Accordingly, the prevalence of using e-cigarettes has been steadily increasing. The United States FDA reported around ten-fold increase in the use of e-cigarettes among high school (from 1.5% to 16.0%) and middle school (from 0.6% to 5.3%) students in the United States during a 4-year period since 2011 [12]. The use of e-cigarette among youth in Korea has increased from 5% to 9% during the same time interval. A similar pattern has been observed in adults too, however, in a lower percentage of individuals [13,14]. Even in the United Kingdom, Public Health England recommends using e-cigarettes as a cessation aid for smoking [15].

However, recent epidemiological evidences suggested that exposure to even low doses of tobacco increases the risk for health impairments such as lung cancer, and the overall death rate. Some studies have suggested that nicotine itself has harmful effects and stimulates the expression of some well-known oncogenes, such as epidermal growth factor receptor (EGFR), in cancer cells, which leads to poor prognosis and survival in patients [16–20]. Certainly, being a major receptor tyrosine kinase in many human tissues, the aberrant overexpression, and activation of EGFR has long been implicated in the carcinogenesis of various types of cancer.

Currently, there is a lack of general information on the contents of e-liquid mixtures and the harmful effects of even the low dosage use of e-cigarettes. A study is, therefore, necessary to understand the effects of using e-cigarettes. Thus, the present study was conducted to reveal that e-liquid mixture can boost proliferation and malignancy of brain tumor cells by increasing EGFR phosphorylation, resulting in poor prognosis in an orthotopic animal model.

## Methods

### E-cigarette liquid materials

We selected a prevailing Prime nico10 mild e-liquid (Prime Korea, Republic of Korea, http://primejuice.kr/shopinfo/company.html) product in the Korean E-cigarette market based on the development of an analytical method and survey for hazardous substances in liquid phases of new types of electric cigarettes (Centers for disease control and prevention report, Republic of Korea, 11-13520000-001567-01). Major compounds of Prime nico10 mild e-liquid are combined congener, glycerin, propylene glycol, and nicotine (9.5 mg/ml, extracted from tobacco stems).

### Cell culture and e-liquid treatment

CSC2 patient-derived glioblastoma stem-like cells (GSCs) were cultured in Dulbecco’s Modified Eagle Medium supplemented with F-12 medium. B27 (**ng/ml), EGFR (ng/ml) and bFGF (ng/ml) supplements, and 1% P/S (ng/ml) were added. Astrocytes were cultured in Dulbecco’s Modified Eagle Medium supplemented with 10% fetal bovine serum and 1% P/S (ng/ml). Cultured cells were incubated at 37°C with 5% carbon dioxide. For western blotting, the cells were cultured as follows: cells were seeded at a density of 1×10^6^ cells per well in a 60ml×15mm petri dish for 48 h before e-liquid treatment. For e-liquid treatment, 1 μL e-liquid (Prime juice) was diluted in 1 mL culture medium and then a volume of 12.5 μL(19.7 ng/ml nicotine concentration), 25 μL(39.5 ng/ml nicotine concentration), and 50 μL(79.1 ng/ml nicotine concentration) per well was added to each respective well for 24 hours. For the vehicle treatment, we have used propylene glycol(Sigma-Aldrich, USA), the major substance described by the company and another researches [21,22]. 1 μL propylene glycol was diluted in 1mL culture media, and 25 μL of this mixture was added to each well for 24 hours. For the astrocyte only vehicle and 25 μL(39.5 ng/ml nicotine concentration) were used. All CSC2 tumor cells and astrocytes used in the experiments were within 5–30 passages (years 2016–2018). CSC2 cell line was a gift from Dr. Myung-Jin Park (Korea Institute of Radiological and Medical Science, Republic of Korea).

### Western blot

Proteins were extracted using RIPA buffer containing a protease inhibitor cocktail (Roche, Switzerland) and a phosphatase inhibitor (Thermo Scientific, USA). Total proteins were separated by electrophoresis and transferred onto PVDF membrane (Millipore, USA) which was blocked with 5% skim milk (BD Biosciences, USA). Protein blots were incubated at 4°C overnight with primary antibodies against EGFR (Cell Signaling Technology, USA) and downstream effector antibodies: Mitogen-activated protein kinase (ERK1/2; Cell Signaling Technology, USA), Signal transducer and activator of transcription 3 (STAT3; Santa Cruz Biotechnology, USA) and Protein kinase B (AKT; Cell Signaling Technology, USA) which are induced by EGFR. Immunoreactive bands were detected using peroxidase-labeled affinity purified secondary antibodies (KPL; ELITechGroup, France) and developed using Miracle-Star western blot detection system (Intron Biotechnology, Republic of Korea) and Amersham ECL prime western blotting detection reagent (GE Healthcare, USA).

### Proliferation assay

Cell proliferation was measured using ATP-based Cell Titer-Glo® Luminescent Cell Viability Assay (Promega, USA). After treating cells with the e-liquid for 24 hours, 500 CSC2 tumor cells were added to each well of the 96-well culture plate and incubated at room temperature in a 1:1 ratio of culture media and the assay reagent. 25 μL (39.5 ng/ml nicotine concentration) of e-liquid and a vehicle were assayed in triplicate, and luminescence was measured by Molecular Devices SpectraMax microplate reader at 0 day, 3 day and 5 day.

### Limiting dilution assay

For *in vitro* limiting dilution assay (LDA), GSCs with decreasing cell concentration (50 cells/well, 25 cells/well, 10 cells/well, and 5 cells/well) were plated in a 96-well plate containing DMEM/F-12 supplemented with B27, epidermal growth factor (EGF, 10 ng/mL), and basic fibroblast growth factor (bFGF, 5 ng/mL). Limiting dilution analysis was performed using the ELDA software (available at http://bioinf.wehi.edu.au/software/elda/).

### Orthotopic mouse model

This study was reviewed and approved by the Institutional Animal Care and Use Committee (IACUC) of the National Cancer Center research institute. NCCRI is an Association for Assessment and Accreditation of Laboratory Animal Care International (AAALAC International) accredited facility and abide by the Institute of Laboratory Animal Resources (ILAR) guide. Approved number: NCC-17-402. For the mouse Temperature and humidity are set to 22±1°C and 50±20%, respectively, in terms of range and are recorded every ten minutes. A sensor is installed in each animal room. If the temperature exceeds 25°C,the designated individuals are notified via mobile phone by SMS. A total of four cooling units, four air handling units, and a central heating system are operated at the temperature and humidity that were automatically set. The operation is monitored 24 hours a day by a central monitoring system. All mice were maintained in an individually ventilated cage (IVC) rack (Threeshine, Republic of Korea), food (Altromin, Germany), autoclaved reverse osmosis (RO) water was provided. We anesthetized mice using Zoletil 50 (06516 Carros, France) to perform brain orthotopic injection. CSC2 tumor cells were detached from the cell culture plates and resuspended in DMEM/F-12 medium supplemented with B27, EGF (10 ng/mL), and bFGF (5 ng/mL). Cells were stereotactically injected into the left striatum of 5-week-old 10 female BALB/c nude mice (Orient Bio Inc., Republic of Korea). The brain injection coordinates were set to 2.2 mm to the left of the midline and 0.2 mm posterior to the bregma at a depth of 3.5 mm [23–25]. The mouse was sacrificed using spinal dislocation. Many studies investigated the toxicity of tobacco and e-cigarette using oral administration or subcutaneous injection for their in-vivo experiments [26–35]. Therefore we performed oral administration of e-liquid to test its carcinogenic role. E-liquid solution was prepared by diluting 1.2 mL e-liquid in 13 mL distilled water and its corresponding vehicle solution was prepared by diluting 1.2 mL propylene glycol into 13 mL distilled water. After 3 days from the orthotopic injection, 300 μL of e-liquid, (103μg/ml nicotine concentration) (6 mg/kg), or vehicle solution was orally fed to the mice daily. After 20 days from the injection, a mouse from each group was sacrificed to perform in-vivo histological studies. Total 4 mice were sacrificed at the endpoint, and 4 mice were found dead. The brains of the sacrificed and dead mice were harvested and fixed in 4% paraformaldehyde. National Cancer Center animal experiment department provides daily animal care and monitoring services. All mice reaching endpoint criteria were sacrificed using spinal dislocation. A weight loss of 10–15% within a few days was a criterion for euthanasia. An overall weight loss of 20% was also an indication of euthanasia. The animal experiment lasted for 41 days after injection. Survival was analyzed with 4 mice in the vehicle group and 4 mice in e-liquid group using GraphPad PRISM software (version 7).

### Magnetic Resonance Imaging (MRI)

MRI analysis was performed, and images were acquired using a BioSpin 7.0 T magnet (Bruker Corporation, Germany). After localized imaging on three orthogonal axes, T2-weighted images of the entire mouse brain were acquired using a Rapid Acquisition with Refocused Echoes (RARE) sequence. The repetition time (TR) and echo time (TE) were adjusted to 2500 ms and 35 ms, respectively. A 2-cm field of view and a 256 × 256 matrix with four averages to achieve a total scan time of 4 min were other parameters that were used in the experiment.

### Hematoxylin and eosin (H&E) staining

H&E staining of the whole brains from mice implanted with CSC2 cells. Scale bar, 100 μm. The sample was extracted at 20 and 41 days after cell injection. To observe histological features, the brain was removed, fixed with 4% paraformaldehyde for 24 h at 4°C, and stained with hematoxylin (DaKo, Germany) and 0.25% eosin (Merck, USA).

### Immunohistochemical (IHC) staining

For immunohistochemical staining of EGFR marker, tissue sections were antigen retrieved with citrate buffer (pH 6.0) followed by endogenous peroxidase blocking with 3% hydrogen peroxide. Further, tissue sections were incubated with primary antibody for pEGFR (Cell Signaling Technology, USA) overnight at 4°C in a humidified chamber. To avoid nonspecific binding in mouse tissue, we used Mouse Elite Peroxidase kit (Vector Laboratories, USA) and developed the sections using 3,3ʹ-diaminobenzidine (DAB; Vector Laboratories, USA) as a chromogen.

### Immunofluorescence (IF) staining

For immunofluorescence staining, mouse brain tissue was sectioned on coated slides. The mouse brain was fixed with 4% paraformaldehyde for 24 h at 4°C and embedded in a paraffin block. The brain tissue section was stained with pERK primary antibody (diluted in blocking solution, 1:100) for 24 h at 4°C and washed three times with PBS. Staining was detected using Alexa Fluor 568 anti-mouse secondary antibody (1:500; Invitrogen, USA) in dark for 1 h at 4°C and washed three times with PBS. Nuclei were counterstained using 4ʹ,6-diamidino-2-phenylindole (DAPI). Stained mouse brain tissues were mounted with a coverslip. Images were captured using confocal microscope (LSM 710, Zeiss, Germany).

### REMBRANDT database analysis

Patients’ survival data based on EGFR expression levels in all gliomas, glioblastoma, and astrocytoma (n = 330) was obtained from the REMBRANDT database of the National Cancer Institute (now available on Georgetown Database of Cancer [GDOC]). There were 185 low EGFR expressing patients (EGFR < 8.965-fold) and 155 high EGFR expressing patients (EGFR > 8.965-fold). Kaplan–Meier survival analysis was used to estimate the survival distributions. The log-rank test was used to assess the statistical significance between the stratified survival groups using Graphpad Prism six statistical software.

### Statistical analysis

Results of multi-dataset mouse experiments were compared by analysis of variance using the log-rank (Mantel-Cox survival curve) test. The results of the two-dataset experiments were compared using a two-tailed Student *t*-test. P < 0.05 or P < 0.01 was considered statistically significant.

## Results

### Elevated level of EGFR expression is associated with poor survival in glioma patients

**Fig 1:**
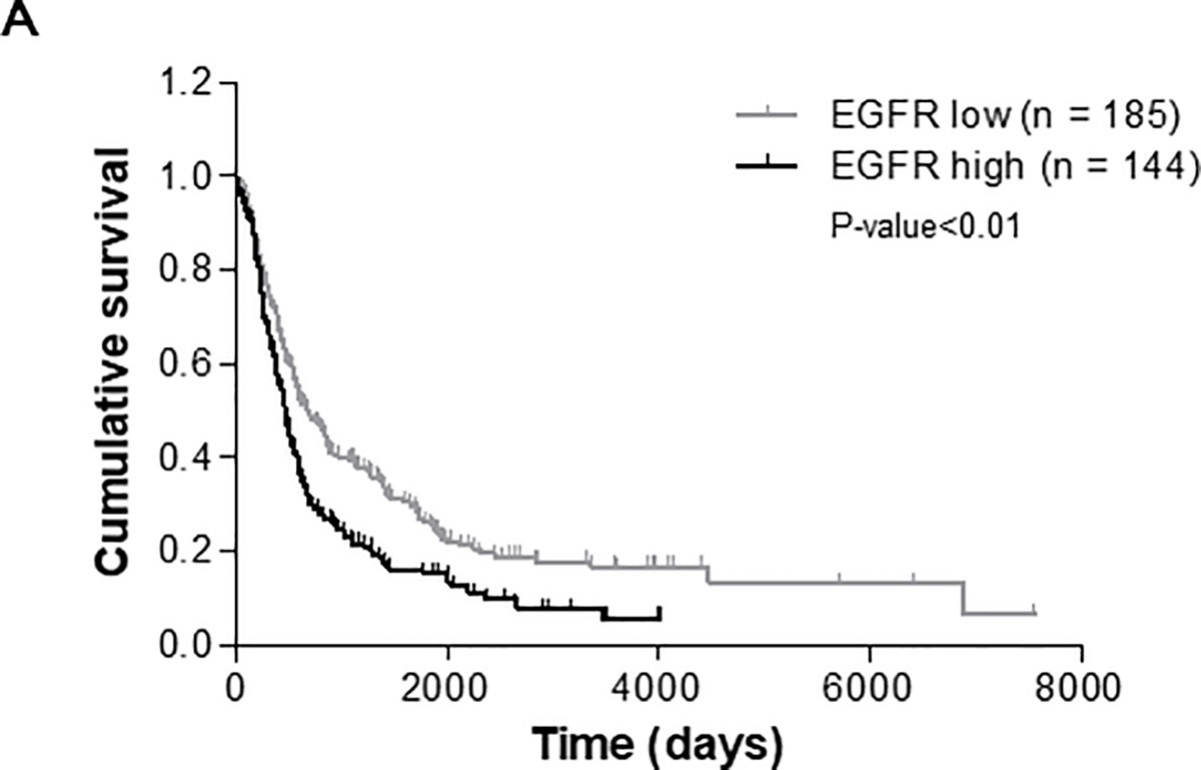
Kaplan-Meier plot of survival curve for all glioma patients according to EGFR expression. (A) These data are based on the log-rank test (P-value<0.01). Data were obtained from the Repository for Molecular Brain Neoplasia Data (REMBRANDT) program of the National Cancer Institute.

EGFR is observed to be overexpressed in 30–40% of higher grade glioma patients. Thus, we first investigated the relationship between EGFR expression and the survival of glioma patients using the REMBRANDT database of the United States National Cancer Institute. The Kaplan-Meier survival plot showed that the EGFR mRNA level was negatively correlated with patient survival (Fig 1A) (P-value<0.01). The glioma patient group with high EGFR expression showed poor prognosis compared to the glioma group with low EGFR expression.

### E-liquid promotes EGFR activation in brain cancer stem cells

**Fig 2:**
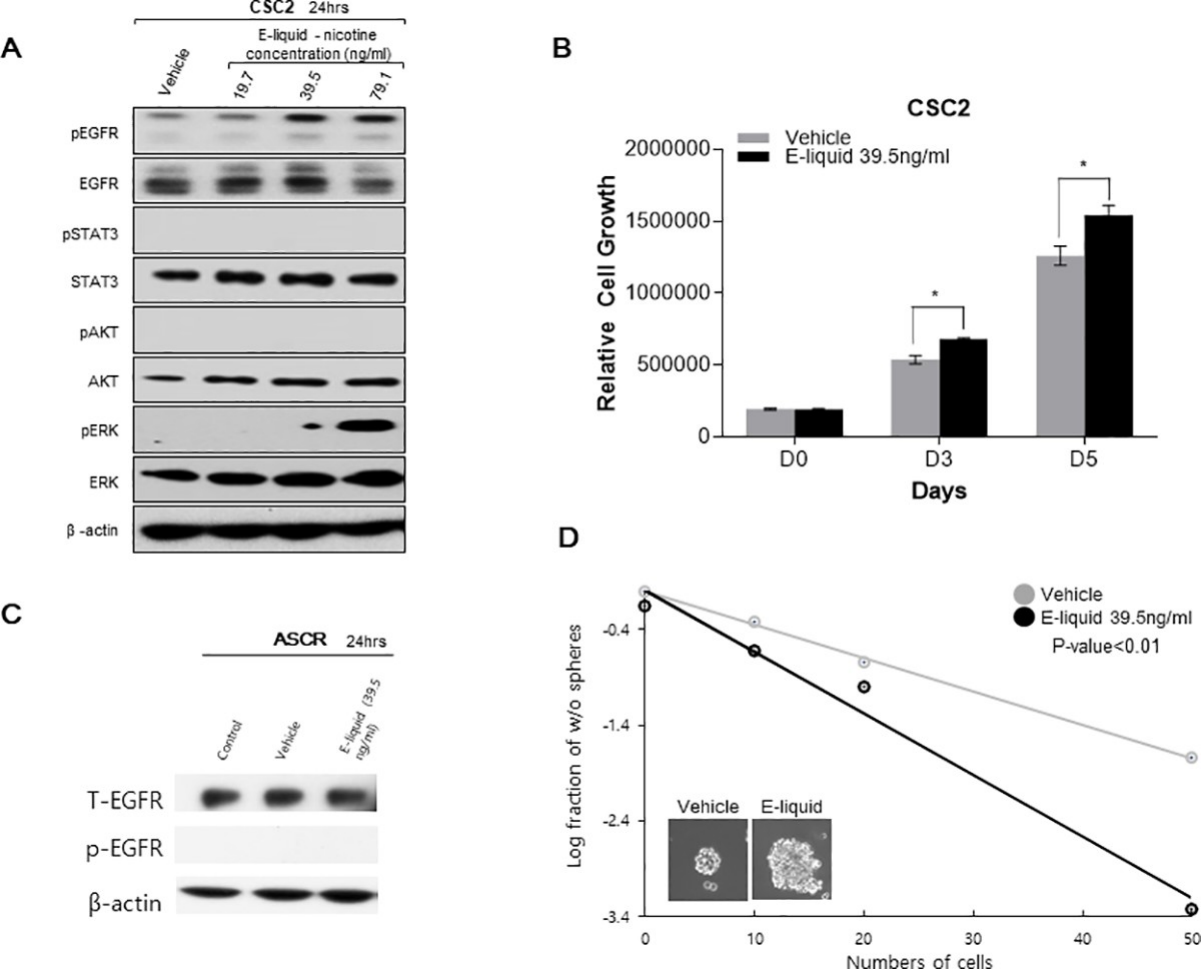
E-cigarette promotes EGFR activation in glioma stem cells (GSCs). (A) Western Blot analysis of pEGFR, EGFR, pERK and ERK in GSCs (CSC2 cells) treated with e-liquid or its vehicle. β-actin was used as a loading. (B) Cell viability assays of GBM patient-derived cells compared with control and e-liquid treatment concentrations of CSC2 (P-value<0.05). (C) Western Blot analysis of pEGFR, EGFR, pERK and ERK in human astrocytes treated with e-liquid or its vehicle. β-actin was used as a loading. (D) Limiting dilution assay of GBM patient-derived cell (CSC2 cells) treated with e-liquid or its vehicle. (P-value<0.01).

Since EGFR overexpression is a frequent and critical event in glioma progression and its activation leads to malignancy in many tumor types, we hypothesized that exposure to e-liquid could aggravate EGFR activation in glioma. Thus, we studied the effect of e-liquid on EGFR expression level. Treating CSC2 with e-liquid showed an increase in pEGFR level compared to the vehicle. We also evaluated the downstream effectors of the canonical EGFR signaling pathway, which included STAT3, Akt, and ERK. We did not observe any changes in pSTAT3 and pAkt expression. Interestingly, the pERK level was increased in e-liquid cells compared to the vehicle cells. The data suggested that e-liquid activates specific transducers of EGFR signaling, which influence the growth of brain cancer stem cells via the activation of the ERK pathway (Fig 2A). Surprisingly e-liquid treatment on human astrocytes, which does not express basal p-EGFR levels, did not affect the expression of pEGFR, and EGFR levels. This observation shows that e-liquid sensitizes already existing p-EGFR levels, which makes GBM patient derived stem cells more susceptible to the use of e-liquid (Fig 2C).

### E-liquid treatment increases proliferation of brain cancer stem cells

As the EGFR-ERK pathway was observed to be activated by e-liquid, we further speculated that e-liquid treatment may also increase cell proliferation. On treating patient-derived CSC2 glioblastoma stem-like cells with e-liquid, we found that the proliferation of CSC2 tumor cells increased compared to the growth rate of the vehicle cells. This indicated that e-liquid had a direct effect on promoting cancer cell growth (Fig 2B). (P-value<0.05).

### E-liquid treatment promotes stemness of brain cancer stem cells

Another key factor determining the malignancy of glioblastoma is the stemness of a small population of tumor cells that harbor a function, which is similar to that of stem cells. In order to identify the effect of e-liquid on the stemness of GSCs, we performed a LDA assay and observed that the group treated with 39.5 ng/ml nicotine concentration of e-liquid had higher sphere forming ability as well as larger size of individual spheres compared to those of the control group (Fig 2D) (P-value<0.01). Our results suggested that e-liquid induces cell proliferation and self-renewal capability in GSCs, thereby promoting tumorigenesis.

### MRI scanning of glioblastoma tissues of orthotopic mouse models

**Fig 3:**
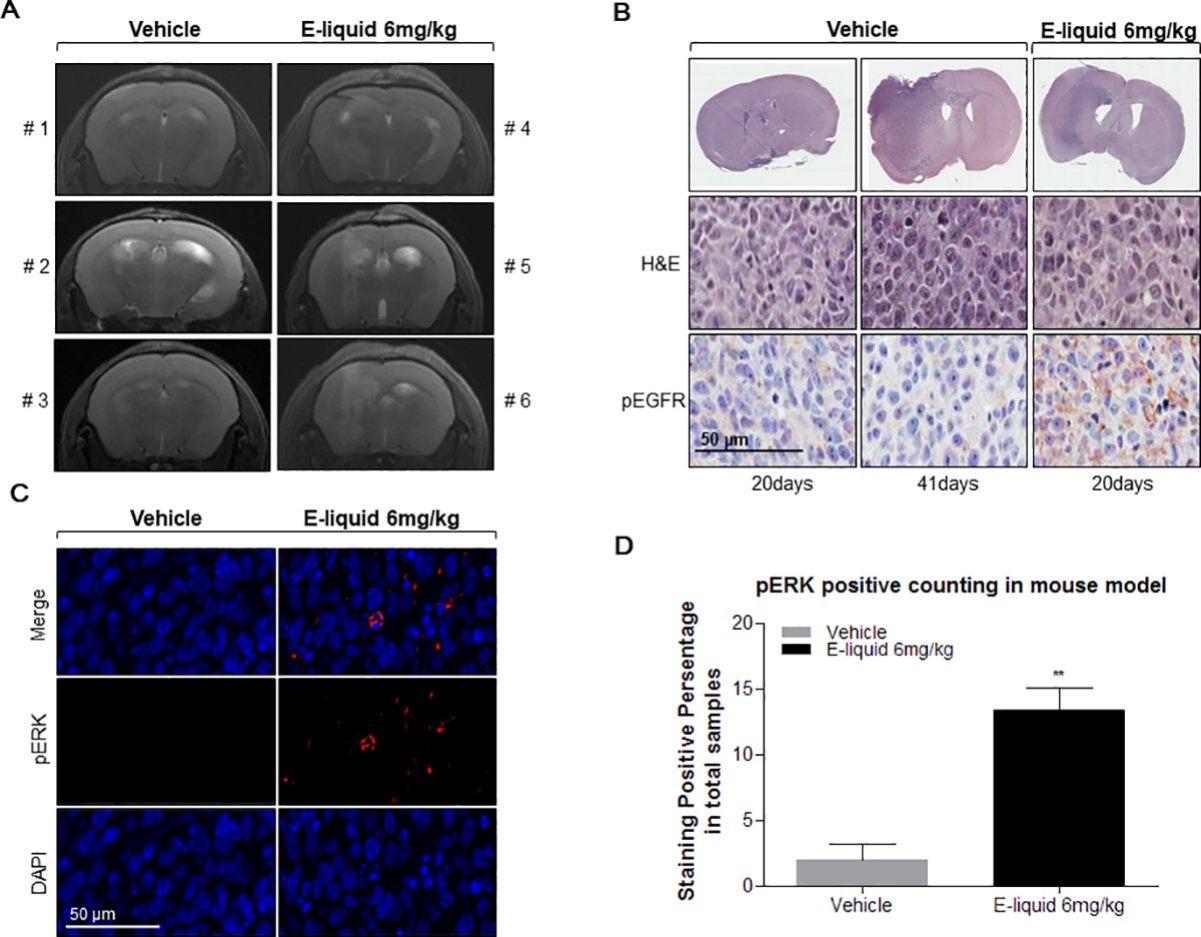
E-liquid activates EGFR signaling and it influenced on growth of brain tumor cell by downstream of pERK in the orthotopic xenograft mouse model. (A) Magnetic Resonance Imaging (MRI) after 21 days of brain injection with CSC2 cells and treated by vehicle treatment (n = 3, 5×10^4^ cells injected in each mouse) and e-liquid treatment (n = 3, 5×10^4^ cells injected in each mouse). (B) Hematoxylin and eosin (H&E) staining of the whole brain. Histopathology of Balb-c/nude mouse brain, orthotopically injected with 5×10^4^ CSC2 cells and treated by e-liquid and vehicle treatment. H&E staining of the whole brain (Upper panel). Immunohistochemical (IHC) stainning analysis of pEGFR in the orthotopic xenograft mouse model (Bottom panel).; all images were taken at 40x magnification. Scale bar, 100 μm (C) Immunofluorochemistry (IFC) staining Histopathology of Balb-c/nude mouse brain, orthotopically injected with 5×10^4^ CSC2 cells and treated by vehicle treatment (left) or E-liquid treatment (right). Upper panel is merge of brain. pERK (middle) DAPI (bottom); all images were taken at 40x magnification, Scale bar, 100 μm (D) pERK positive cell counted percentage of CSC2 cells injected in brain orthotopic mouse model tissues treated with e-liquid or vehicle (P-value<0.01).

To further validate the phenotype, we generated orthotopic mouse models using the CSC2 tumor cell line. To compare tumor size between the vehicle and e-liquid treated groups, we captured MRI images of mice after 21 days of injecting the brain with CSC2 cells and 18 days of treating with e-liquid and vehicle treatment. Imaging was performed with 3 mice each from the vehicle and e-liquid groups, at the same time point. The e-liquid group was observed to have accelerated tumor growth compared to that of the vehicle group at the given time point (Fig 3A).

### E-liquid treatment promotes tumor growth

To assess the tumorigenic role of e-liquid on glioblastoma stem-like cells, we extracted brain sample from each group, 20 days after orthotopic injection of CSC2 tumor cells. We observed that the mouse from the vehicle group did not have induced brain tumor formation within 1 month of CSC2 cell injection. On the contrary, the mouse from the e-liquid treated group developed brain tumors and showed a highly infiltrative phenotype (Fig 3B). The result showed that e-liquid confers higher activation of pEGFR in brain tumor, which contributes to rapid tumor growth.

### E-liquid treatment increases pEGFR levels in *in vivo* model

To understand the functional significance of e-liquid on CSC2 cell injected orthotopic model, we extracted brain from mice at the end of observation time point (when tumor was formed): 41 days for vehicle group mouse and 20 days for e-liquid group mouse after CSC2 cell injection. Although the vehicle as well as e-liquid treated mouse developed brain tumor by the end time point, pEGFR expression in e-liquid treated mouse was found to be higher compared to the expression levels in vehicle treated mouse (Fig 3B).

### E-liquid treatment increases pERK level

Since we observed increase in pEGFR expression on e-liquid treatment in the orthotopic mouse model, we investigated the downstream signal transducers through immunofluorescence staining. The brains of both the groups were checked for tumor formation and extracted at the end time point (41 days for vehicle group and 20 days for e-liquid group). Consistent to pEGFR, it was observed that pERK expression was higher in e-liquid treated mice compared to its expression levels in vehicle treated mice (Fig 3C) (P-value<0.01). From the obtained data, we concluded that elevated activation level of EGFR stimulates the increase in pERK expression related downstream signaling pathway which may be responsible in promoting malignancy of brain tumor in the orthotopic mouse models.

### Kaplan-Meier curve of orthotopic mouse model

**Fig 4:**
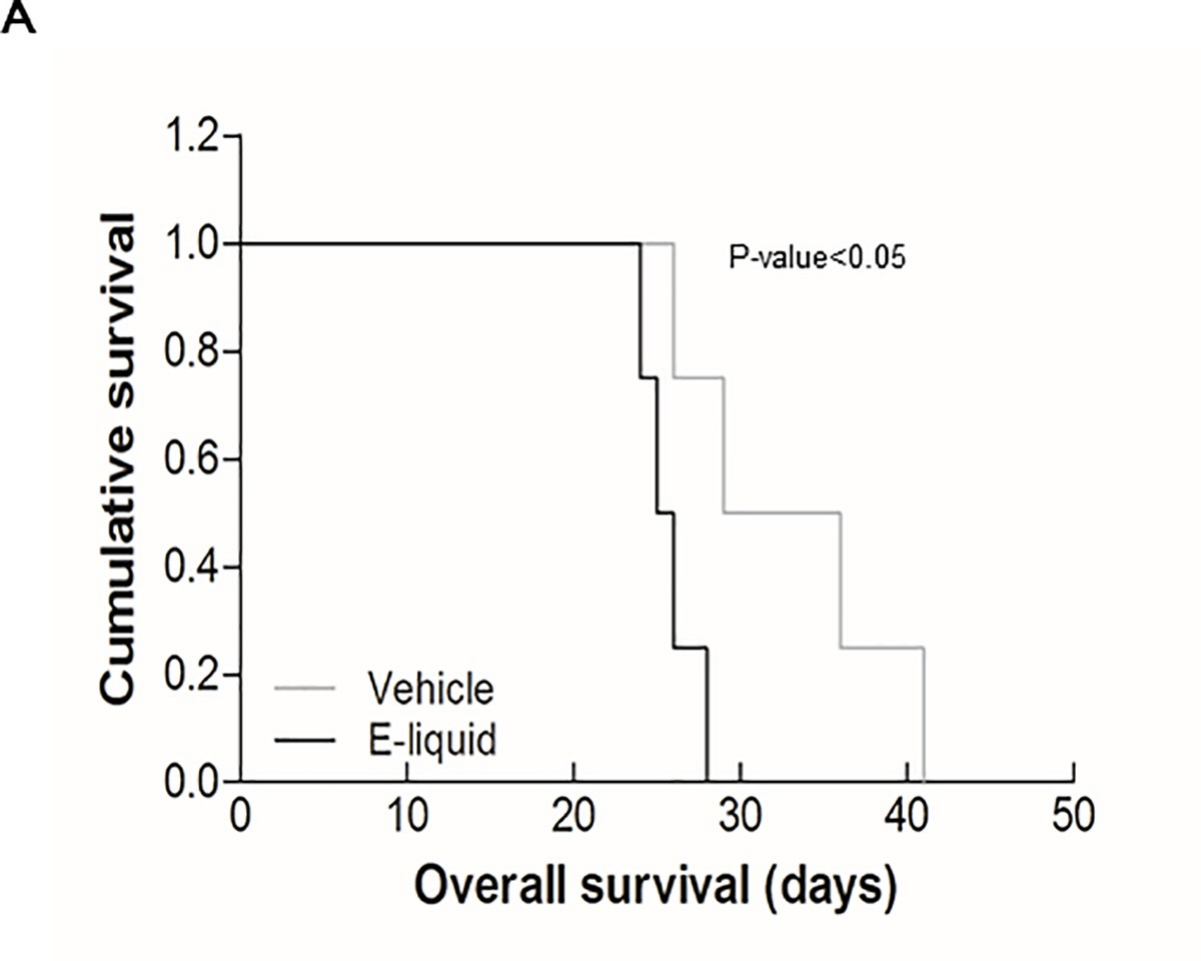
(A) Kaplan–Meier survival graph shows e-liquid group had poor prognosis compared to the vehicle group (P-value<0.05). Kaplan–Meier survival graph of mice implanted with CSC2 cells and treated by vehicle treatment (n = 4, 5×10^4^ cells injected in each mouse). And e-liquid treatment (n = 4, 5×10^4^ cells injected in each mouse).

We generated a Kaplan-Meier curve to compare the survival rates of the orthotopic mouse models. Brain tumor tissues with pEGFR expression showed poor prognosis compared to that of the vehicle group upon e-liquid treatment (Fig 4). Median survival was found to be 32.5 days for the vehicle group, whereas it was found to be 25.5 days for the e-liquid group. (P-value<0.05) The results confirmed the additive effect of e-liquid on brain tumor malignancy.

## Discussion

We were able to establish the relation between e-liquid and EGFR expression in brain tumor. EGFR is a major player in one of the most important cancer growth related pathways. Through an *in vitro* study, we revealed the significance of the effect of e-liquid on brain cancer by confirming a direct relationship between e-liquid treatment and EGFR signal activation. In addition, we showed that the increased level of EGFR phosphorylation in brain cancer cells is due to e-liquid exposure. As a result, total EGFR activation remains relatively constant under all conditions; however, pEGFR expression level increases upon exposure to e-liquid in a dose dependent manner. To further understand the signal transduction, we investigated the expression level of well-known downstream effectors of the EGFR signaling pathway: pSTAT3, pAKT, and pERK. We found that there was no change in the expression of pSTAT3 and pAKT; however, pERK expression increased in a dose dependent manner. Thus, we conclude that e-liquid promotes brain tumor cell proliferation via the activation of the EGFR-pERK signaling pathway.

To further validate the findings of the *in vitro* experiment, we conducted a mouse model experiment by injecting CSC2 brain tumor cells in 10 BALB/c nude mice which were randomly divided into vehicle group (n = 5) and e-liquid treated group (n = 5). MRI and H&E staining showed that e-liquid treated group showed accelerated tumor growth compared to the tumor growth rate observed in the vehicle group. Furthermore, brain tumor formation was seen within 20 days in e-liquid treated group compared to more than 1 month in the vehicle mouse group. There was also an increased expression of activated pEGFR in the e-liquid treated group. We compared the survival rates of mice by Kaplan–Meier survival curve. Higher expression of pEGFR on e-liquid treatment significantly decreased mice survival rate compared to the survival rate of the vehicle group. Thus, the *in vitro* cell study showed that e-liquid activates EGFR signaling and influences the growth of brain tumor cells via pERK activation while animal xenograft study showed that e-liquid promotes accelerated cancer growth which leads to poor prognosis. One point to note is that the current experiment setting is an artificial condition and the findings may not directly reflect the real human vaporizing condition. Although there is a discrepancy between the route of e-liquid administration in the experimental setting and real human vaporizing condition, either way, e-liquid phosphorylates EGFR and ERK pathways through the blood system, which we expect that our observations should reflect the consequences of the actual human condition.

Overall, the present study contributes to the existing literature by showing that e-liquid exposure can boost cell proliferation and malignancy of brain tumor via the increase in EGFR phosphorylation. Such mechanism may be induced in other types of cancers as well. Even though the study does not highlight cancer development, it provides information that can help advise cancer patients who decide to use e-cigarette regarding their potentially harmful effects on cancer progression. For further investigations, experiments using nicotine receptor antagonists are needed to verify the specific consequence of e-liquid treatment after removing nicotine from the mixture. Moreover, long time e-liquid treatments in normal cells need to be considered in order to understand the potential mechanism through which e-liquid contributes to the development of cancer. Due to limited epidemiological studies relating to the usage of e-cigarette and the incidence and survival of brain tumor, further population based studies are needed to confirm our results in humans.

## Supporting information

S1 Raw images (PDF)

S2 Raw images (XLSX)

S3 Raw images (XLSX)

## Abbreviations

DAPI 4ʹ: 6-diamidino-2-phenylindole
EGFR: Epidermal growth factor receptor
e-cigarettes: Electric cigarettes
e-liquid: E-cigarette liquid
TSNAs: Tobacco-specific nitrosamines; group 1 carcinogen
GSCs: Glioblastoma stem-like cells
H&E: Hematoxylin and eosin
IHC: Immunohistochemical
IF: Immunofluorescence
LDA: Limiting dilution assay
mRNA: Messenger RNA
pEGFR: Phosphorylated-EGFR
pERK: Phosphorylated-ERK
pSTAT3: Phosphorylated-STAT3
pAKT: Phosphorylated-AKT
