# Supplementary material for: Analysis of electric cigarette liquid effect on mouse brain tumor growth through EGFR and ERK activation
Source: PLoS One. 2021 Sep 8;16(9):e0256730. doi: 10.1371/journal.pone.0256730 (PMC8425573; doi:10.1371/journal.pone.0256730)
Supplement: S1 Raw images — (PDF) [file pone.0256730.s001.pdf]

# Western blot data

# CSC2 T-EGFR, p-EGFR, B-actin (Low exposure)

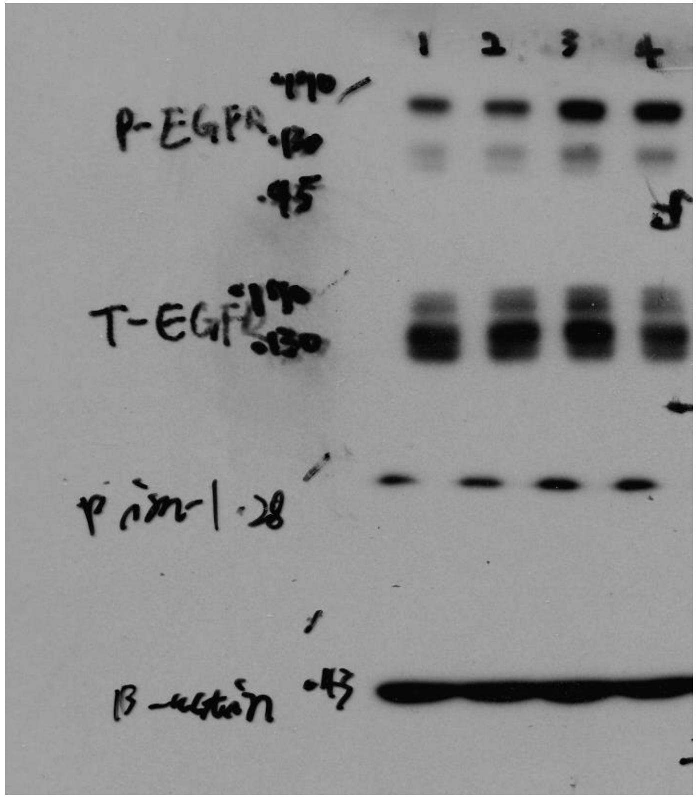


Nicotine concentration

1=Vehicle, 2=19.7 ng/ml, 3=39.5 ng/ml, 4=79.1 ng/ml

# CSC2 T-STAT3, p-STAT3, T-AKT, p-AKT, T-ERK, p-ERK (Low exposure)

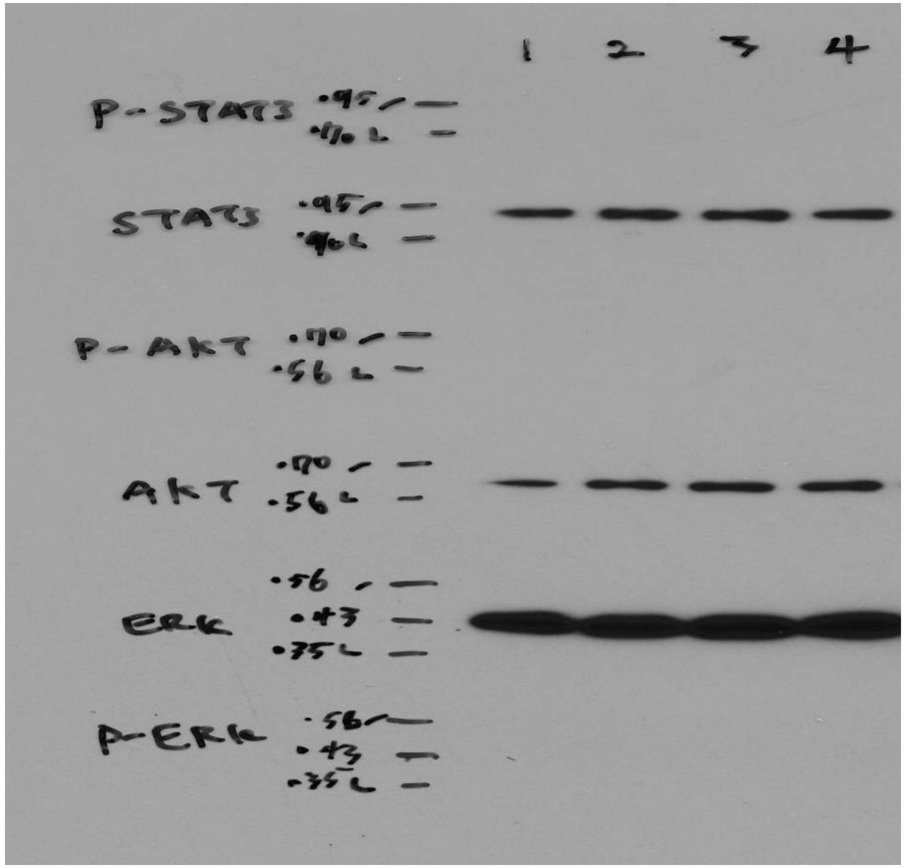


Nicotine concentration

1=Vehicle, 2=19.7 ng/ml, 3=39.5 ng/ml, 4=79.1 ng/ml

# CSC2 p-STAT3, p-AKT, p-ERK (High exposure)

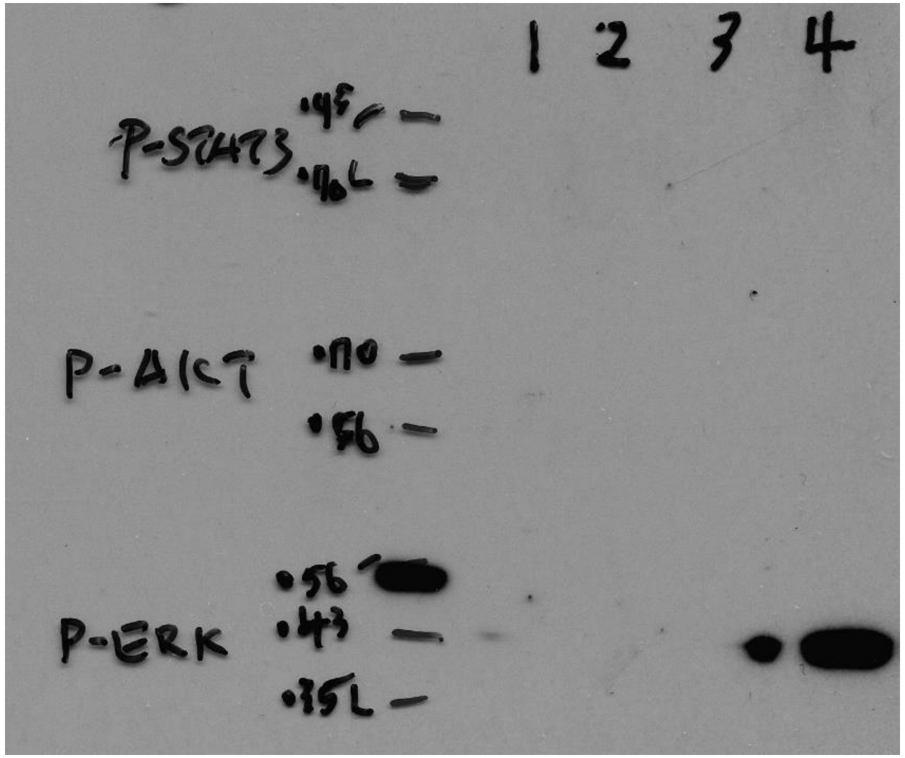


Nicotine concentration

1=Vehicle, 2=19.7 ng/ml, 3=39.5 ng/ml, 4=79.1 ng/ml

# ASCR T-EGFR, p-EGFR, B-actin (Low exposure)

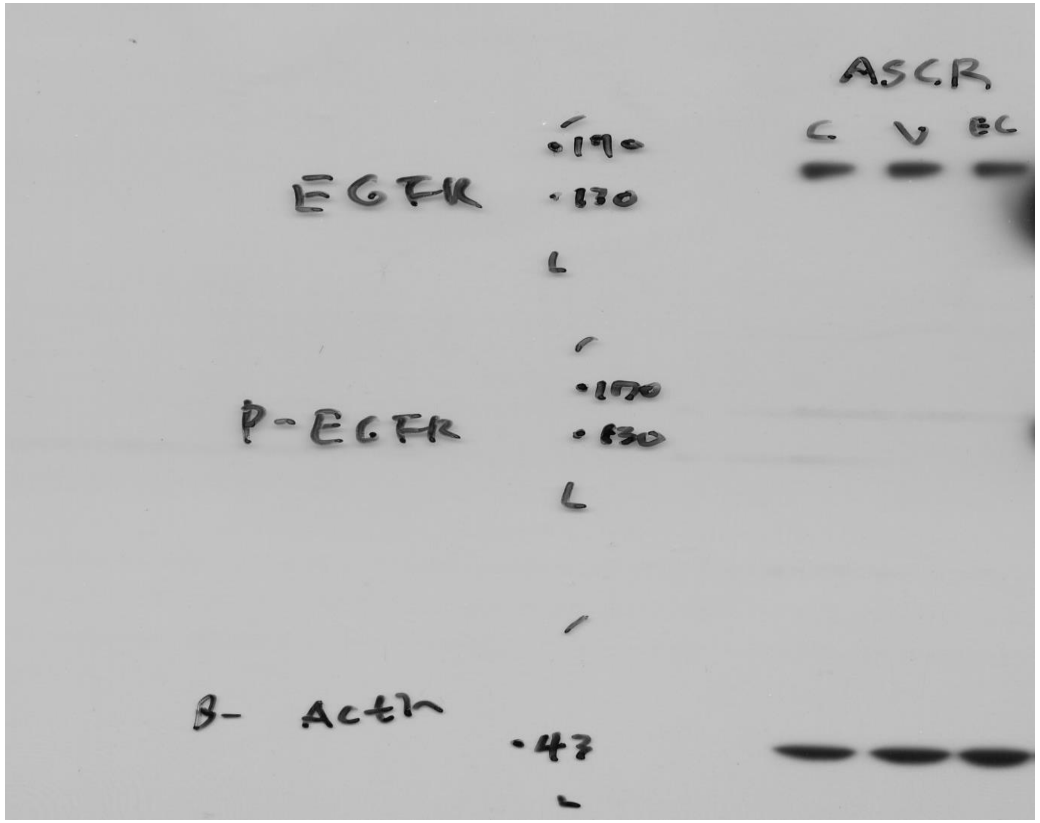


Nicotine concentration

C=Control, V=Vehicle, EC=39.5 ng/ml
